# Cost and cost-effectiveness of adjuvant trastuzumab in the real world setting: A study of the Southeast Netherlands Breast Cancer Consortium

**DOI:** 10.18632/oncotarget.16985

**Published:** 2017-04-09

**Authors:** Shanly C. Seferina, Bram L.T. Ramaekers, Maaike de Boer, M. Wouter Dercksen, Franchette van den Berkmortel, Roel J.W. van Kampen, Agnès J. van de Wouw, Adri C. Voogd, Vivianne C.G. Tjan Heijnen, Manuela A. Joore

**Affiliations:** ^1^ Department of Medical Oncology, Maastricht University Medical Center, Maastricht, The Netherlands; ^2^ GROW-School for Oncology and Developmental Biology, Maastricht University Medical Center, Maastricht, The Netherlands; ^3^ Department of Internal Medicine, Máxima Medical Center, Veldhoven, The Netherlands; ^4^ Department of Internal Medicine, Zuyderland Medical Center, Heerlen, The Netherlands; ^5^ Department of Internal Medicine, Zuyderland Medical Center, Sittard-Geleen, The Netherlands; ^6^ Department of Internal Medicine, VieCuri Medical Center, Venlo, The Netherlands; ^7^ Department of Epidemiology, Maastricht University Medical Center, Maastricht, The Netherlands; ^8^ Department of Clinical Epidemiology and Medical Technology Assessment, Maastricht University Medical Center, Maastricht, The Netherlands; ^9^ School for Public Health and Primary Care, Maastricht University Medical Center, The Netherlands

**Keywords:** trastuzumab, cost-effectiveness, early breast cancer, real-world, Markov model

## Abstract

**Background:**

We assessed the real world costs and cost-effectiveness of the addition of trastuzumab in HER2 positive early breast cancer compared to chemotherapy alone in the Dutch daily practice as opposed to the results based on trial data and based on a subset of patients that were treated according to the guidelines.

**Patients and Methods:**

In a cohort study, we included all patients with stage I-III invasive breast cancer treated with curative intent in 5 Dutch hospitals between 2005 and 2007 (*n*=2684).We assessed three scenarios: a real-world scenario, a trial scenario and a guideline scenario, with costs and effectiveness based on either the cohort study, the published trials or the guidelines. Incremental cost-effectiveness ratios (ICERs) and cost-effectiveness acceptability curves (CEACs) were constructed.

**Results:**

Costs were €243,216 and €239,657 for trastuzumab and no trastuzumab for the real world scenario, €224,443 and €218,948 for the guideline scenario and €253,666 and €265,116 for the trial scenario. The QALYs were 0.827, 0.861, 0.993 for the real world, guideline and trial scenario. The corresponding ICERs were €4,304, €6,382 and dominance, respectively. CEACs showed that the probability that trastuzumab is cost-effective is ≥99% in each scenario.

**Conclusion:**

Adjuvant trastuzumab in the real world can be considered cost-effective.

## INTRODUCTION

In the Netherlands, the use of trastuzumab for the adjuvant treatment of patients with HER2-positive early breast cancer was estimated to be €5,828 per quality adjusted life year (QALY) gained (using a health care perspective). We obtained this Dutch estimate of the cost-effectiveness by transferring the results of a UK (commissioned by NICE) model-based cost-effectiveness analysis of trastuzumab in the early breast cancer setting to the Dutch setting. [1] This analysis provided an early indication of the cost-effectiveness of trastuzumab in the adjuvant setting in the Netherlands. Partly based on this analysis, the Dutch Health Care Insurance Board (CVZ) permitted provisional reimbursement of trastuzumab as adjuvant treatment as of 2005. However, although the structure of the UK cost-effectiveness model was deemed transferable, the model inputs appeared to be only partially transferable to the Dutch setting. Continued reimbursement depended on study of prospective data on the cost-effective use of trastuzumab in daily practice within the Netherlands. A number of studies have investigated the cost-effectiveness of trastuzumab in the adjuvant setting. These studies typically found that adjuvant trastuzumab treatment has an acceptable cost-effectiveness ratio. [2-7] However, in most cases estimates of resource use were based on small datasets or even expert opinion. Moreover, these studies were based on the assumption that the effectiveness and, to some extent, resource use in the real world are equal to the ones observed in a trial setting. Since it is known that randomized controlled trials have a strong internal validity but may have limited external validity, there is reason to think that the real world indication (unselected population) and treatment regimens for trastuzumab differ from the trial situations (selected population) and that the effectiveness is likely to be different too. [8].

The primary aim of our analysis was to assess the costs and cost-effectiveness of one year of adjuvant trastuzumab compared to no trastuzumab for HER2 positive breast cancer, given sequentially after chemotherapy in clinical practice (real world) as opposed to the cost-effectiveness of trastuzumab based on trial data and based on a subset of patients that were treated in full concordance with the Dutch clinical guideline.

## PATIENT AND METHODS

### Model description

**Figure 1:**
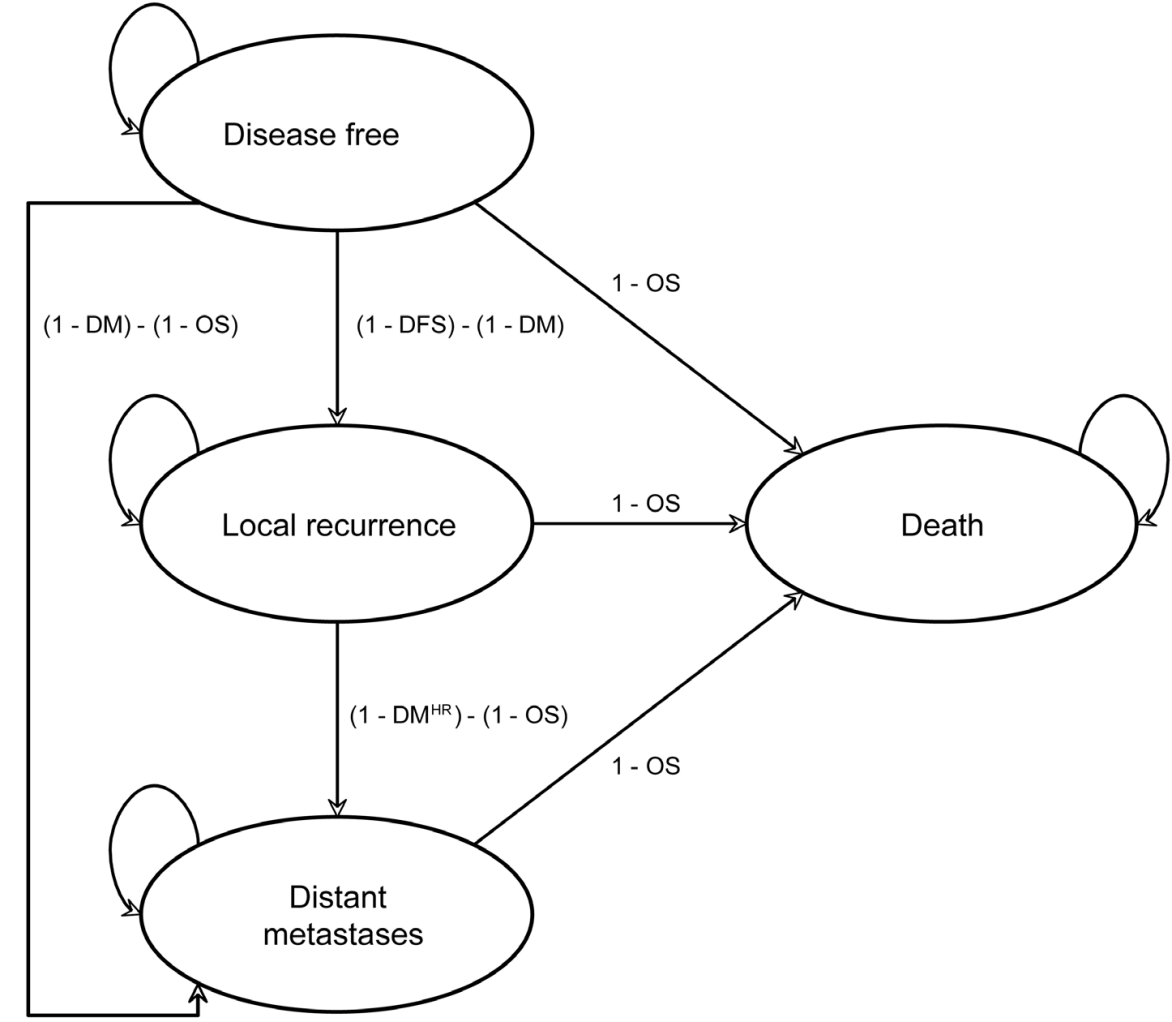
Markov state transition model structure^a^ Abbreviations: OS, overall survival; DFS, disease free survival and; DM, distant metastases free survival; HR, hazard ratio for increased risk of distant metastases after local recurrence. ^a^Note that cardiac events are incorporated during the first year only.

In this study we assessed the costs and cost-effectiveness of one year of adjuvant trastuzumab treatment in early HER2-positive breast cancer *versus* chemotherapy alone. A Markov health state transition cohort model was developed for HER2 positive breast cancer consisting of four health states: (1) disease-free survival (DFS), (2) locoregional recurrence, contralateral recurrence and a new primary tumor (local recurrence, LR), (3) distant metastases (DM), and (4) death (due to cancer and due to other causes). Additionally, in the model cardiac toxicity could occur during the first year (Figure 1). The time horizon of the model was lifetime and the cycle length one year. The analyses were conducted using the Dutch health care perspective.

Transitions in the model were estimated using an exponential survival function (based on Kaplan Meier (KM) curves) for overall survival (OS), DFS and DM. These survival functions were used to calculate the annual probabilities of LR, DM and mortality. The model was used to compare the costs, life years (LYs) and QALYs of treatment with one year of adjuvant trastuzumab sequentially after chemotherapy in patients with HER2-positive early breast cancer *versus* chemotherapy alone. We assessed how the incremental costs of trastuzumab related to differences in LYs and QALYs.

Three scenario analyses were performed, a real world scenario, a trial scenario and a guideline scenario. In all three scenarios, the relative treatment effect of trastuzumab was based on the HERA trial. In addition, in all three scenarios health care resource use other than trastuzumab costs were based on the real world cohort study. The scenarios differed in how the annual probabilities for trastuzumab (OS, DFS, DM-free survival and cardiac toxicity) and the costs of trastuzumab (i.e. number of administrations and dose per patient) were determined.

In the real world scenario annual trastuzumab probabilities for OS, DFS, DM-free survival and cardiac toxicity, and costs associated with trastuzumab were taken from all patients who received trastuzumab in the real world cohort study. [9].

In the guideline scenario annual probabilities for OS, DFS, DM-free survival and cardiac toxicity, and costs associated with trastuzumab were taken from the subgroup of patients in the real world cohort study who received trastuzumab following the criteria listed in the 2005 Dutch clinical guideline*.* [9].

In the trial scenario in which annual probabilities for OS, DFS, DM-free survival and cardiac toxicity were taken from the HERA trial, and costs associated with trastuzumab were partly taken from the HERA trial and partly from the real world cohort study. [10-13].

The model was built and analyzed in Microsoft Excel. An overview of the sources of the input parameters in the three scenario analyses is provided in Table 1.

**Table 1:** The data sources used for parameters in the real world, guideline and trial scenario

Scenarios	Real world	Guideline	Trial^
Disease-free survival	Cohort*	Cohort**	HERA trial
Overall survival	Cohort*	Cohort**	HERA trial
Distant metastases free survival	Cohort*	Cohort**	HERA trial
HR Disease-free Survival	HERA trial		
HR Overall survival	HERA trial		
HR Distant metastases	HERA trial		
Duration of treatment benefit	HERA trial		
Probability cardiac event trastuzumab	Cohort*	Cohort*	HERA trial
RR^#^ cardiac event trastuzumab	HERA trial		
Resource use (other than trastuzumab)	Cohort*		
Costs associated with trastuzumab	Cohort*	Cohort**	Cohort & HERA trial
Health state utilities	Cohort*		

^The HERA trial results are published in [10-14]. * Including all HER2 positive patients; ** Patients who received trastuzumab according to the Dutch 2005 guideline.# Relative Risk.

### Model input parameters

#### Real world cohort study

Detailed information on patient and tumor characteristics are reported elsewhere. [9].

In brief, we included 2684 patients with consecutive diagnosis of stage I-III breast cancer in the five participating hospitals between January, 1st 2005 and December 31st, 2007. Follow-up was collected until October 20th 2011. Data were collected by trained data managers under direct supervision of medical specialists and researchers. For patients of whom the HER2 status was missing, central pathology review was performed. In total, 476 patients had a HER2 positive tumor. Patients were classified in a cohort of patients who received trastuzumab (*n* = 230) and a cohort of patients who did not receive trastuzumab (*n* = 246). In the guideline scenario there were 196 patient who did received trastuzumab and 191 patients who did not receive trastuzumab.

#### Transition probabilities

In the real world scenario, transition probabilities were estimated based on the trastuzumab group in the cohort study. Hazard ratios (HR) were taken from the HERA trial, and applied to the real world exponential survival functions for 8 years, afterwards the HRs were assumed to be 1. HRs for DFS and OS were based on the 8-years follow up data of the HERA trial [13]. The HR for DM-free survival was based on the 2-years follow up data [11] as for this outcome 8-years follow up was not reported. The relative risk (RR) for cardiac toxicity was based on the HERA trial. [14] In the scenario based on the 2005 guideline the same approach was taken, using the subset of patients (N = 196) who received trastuzumab following the Dutch 2005 clinical guideline to derive the transition probabilities. In the trial scenario DFS and OS were based on the 8-years follow up data of both arms of the HERA trial [13]. DM was based on the 2-years follow up data [11] as for this outcome 8-years follow up was not reported. Age related mortality based on Dutch all-cause mortality for women was used to estimate the time-dependent mortality probability for the DFS health state in all three scenarios. [15].

#### Resource use and costs

Health care resource use was based on the real world cohort study. The trastuzumab related costs included trastuzumab treatment, hospital cost when receiving the treatment and cardiac monitoring costs. Also the cost to determine the HER2 status was included, using either immunohistochemistry (IHC) tests, FISH tests, or both. The actual amount of trastuzumab vials used was determined for the calculation of drug costs (taking into account drug spill). The number of cycles, used to calculate the drug costs and costs of administration, was either based on cohort data (real-world and guideline scenario) or based on the HERA trial (trial scenario).

We assumed all other medical resource use was independent from the use of trastuzumab. The medical costs per health state were based on the total hospital resource use as observed among all HER2 positive patients in the real world cohort study. This included surgical procedures, diagnostic procedures, radiotherapy, pharmacological treatments, admissions, outpatient consultations. These were estimated separately for patients with DFS, LR, and DM and separately for the first year and subsequent years. Unit prices for drugs were based on the Dutch reimbursement system for pharmaceuticals. [16] All other unit costs were derived from the national health tariffs authority. [17] Indexation to 2012 price levels was carried out using price index numbers reported by the Central Bureau of Statistics (CBS) in the Netherlands. [15] Total costs per health state are shown in Table 3.

**Table 2:** Transition probabilities used in the model in the three scenarios

Transition probabilities *Real world scenario – trastuzumab*	Estimate	Standard Error	Distribution	Source
1-Overall Survival, first year*	0.000	-	fixed	Cohort
1-Overall Survival , > first year*	0.026	0.006	Beta	Cohort
1-Disease Free Survival, first year	0.016	0.031	Beta	Cohort
1-Disease Free Survival, > first year	0.054	0.067	Beta	Cohort
1-Distant Metastases, first year	0.003	0.002	Beta	Cohort
1-Distant Metastases, > first year	0.041	0.006	Beta	Cohort

Transition probabilities *Guideline scenario - trastuzumab*	Estimate	Standard Error	Distribution	Source
1-Overall Survival, first year*	0.000	-	fixed	Cohort
1-Overall Survival , > first year*	0.029	0.006	Beta	Cohort
1-Disease Free Survival, first year	0.019	0.009	Beta	Cohort
1-Disease Free Survival, > first year	0.052	0.006	Beta	Cohort
1-Distant Metastases, first year	0.004	0.003	Beta	Cohort
1-Distant Metastases, > first year	0.048	0.007	Beta	Cohort

Transition probabilities *Trial scenario – trastuzumab*	Estimate	Standard Error	Distribution	Source
1-Overall Survival, first year*	0.023	0.001	Beta	HERA[13]
1-Overall Survival , > first year*	0.023	0.001	Beta	HERA[13]
1-Disease Free Survival, first year	0.042	0.002	Beta	HERA[13]
1-Disease Free Survival, > first year	0.042	0.002	Beta	HERA[13]
1-Distant Metastases, first year	0.050	0.004	Beta	HERA[11]
1-Distant Metastases, > first year	0.050	0.004	Beta	HERA[11]

Transition probabilities *All scenarios*	Estimate	Standard Error (HR)	Distribution	Source
HR Overall Survival	0.760	0.077	LogNormal	HERA[13]
HR Disease Free Survival	0.760	0.064	LogNormal	HERA[13]
HR Distant Metastases	0.600	0.102	LogNormal	HERA[11]
HR for increased risk of distant metastases after local recurrence	3.640	0.300	LogNormal	HERA[31]
Duration of treatment benefit (years)	8.000	0.307	LogNormal	HERA[13]

*In case OS is smaller than age related mortality based on Dutch all-cause mortality for women, the age related mortality is used

In all three scenarios the HR for the group not treated with trastuzumab was calculated using the inverse of the HR of the HERA trial.

**Table 3:** Total health state costs and utility scores per year

Cost input parameters	Estimate	Standard Error	Distribution	Source
**Health state costs (per year)**				
Disease Free Survival, first year	€12,776	€798.14	Gamma	Cohort
Disease Free Survival, > first year	€1,237	€107.49	Gamma	Cohort
Local recurrence, first year	€12,777	€2,203.85	Gamma	Cohort
Local recurrence, > first year	€14,149	€4,679.22	Gamma	Cohort
Distant Metastases, first year	€30,165	€2,339.46	Gamma	Cohort
Distant Metastases, > first year	€47,959	€13,877.79	Gamma	Cohort
Cardiac Monitoring, first year	€467	€18.90	Gamma	Cohort
Trastuzumab # vials 150 mg per cycle	3	0.050	Gamma	Cohort
Trastuzumab # administrations	15	0.420	Gamma	Cohort
Trastuzumab cost / vial 150 mg	€605	-	Fixed	
Costs of administration (day care)	€257	-	Fixed	

Health state utility scores	Estimate	Standard Error	Distribution	Source
Disease free survival, first year	0.728	0.016	Beta	Cohort
Disease free survival, > first year	0.805	0.021	Beta	Cohort
Local recurrence, first year	0.725	0.021	Beta	Cohort
Local recurrence, > first year	0.708	0.088	Beta	Cohort
Distant metastases, first year	0.584	0.063	Beta	Cohort
Distant metastases, > first year	0.604	0.046	Beta	Cohort
Cardiac toxicity	0.600	0.010	Beta	[19]
Disutility	0.128	0.019		

*treatment costs were assumed to be fixed since differences in these costs are most likely a result of variability (not parameter uncertainty)

#### Health state utilities

Quality of life weights were derived from a cross sectional survey among patients with breast cancer in four of the five medical centers participating in the real world cohort study (*n* = 268). At their regular outpatient visit patients with breast cancer, irrespective of treatment or disease state, were asked to fill out the EQ-5D questionnaire once. The health state utility scores were calculated using the UK tariff developed by Dolan et al. [18] Severe cardiac adverse events were not observed in the patients who participated in the cross sectional survey. Therefore, a utility for symptomatic heart failure (0.600; [19]) was taken from the literature to calculate a disutility of 0.128 ( = 0.728 - 0.600) for severe cardiac adverse events. The utility 0.728 is the mean utility for DFS in the first year after diagnosis from the cohort. See Table 3 for an overview of the utility scores used in the model.

### Analyses

#### Base case analyses

Expected LYs, QALYs and costs (cardiac event/monitoring cost, disease free cost, local recurrence cost and distant metastases cost) were estimated in the three scenarios. Subsequently, the incremental cost-effectiveness ratio (ICER) for each scenario was calculated by dividing the incremental costs by the incremental QALYs. If the ICER is below the maximum amount society is willing to pay per gained QALY, the new treatment can be considered cost-effective. In the Netherlands, a maximum willingness-to-pay threshold of 80,000 euro per gained QALY was proposed for high burden diseases. [20] A half cycle correction was applied for QALYs and health state costs in all three scenarios. Discounting was carried out according to the Dutch guidelines for pharmacoeconomic research, in which future costs and effects are discounted by rates of 4.0% and 1.5%, respectively. [17].

#### One-way sensitivity analysis

Although the 8-years results of the HERA trial still showed a treatment benefit, the impact of a shorter duration of treatment benefit of 4 years was explored in a one-way sensitivity analysis. [13] Moreover, the impact of assuming treatment dependent DM health state cost (instead of treatment independent as in the base case analysis) was explored. Resulting in lower cost for patients treated with trastuzumab *versus* those not treated with trastuzumab.

#### Probabilistic sensitivity analysis and value of information analysis

Probabilistic sensitivity analysis (PSA) was performed to examine the uncertainty in all stochastic input parameters simultaneously. This was done by assigning distributions to the input parameters and drawing random values from these distributions using Monte Carlo simulation with 10,000 iterations. Cost-effectiveness acceptability curves were used to present the results of the PSA. As the results are uncertain, it is possible that the wrong decision is made when implementing the most cost-effective strategy based on the current analysis. The expected value of perfect information (EVPI) analysis can be used to assess the expected costs of this decision uncertainty. In this way, the EVPI can be interpreted as the maximum that society should be willing to pay for further evidence to reduce this decision uncertainty. [21] The population EVPI was calculated by multiplying the EVPI per patient by the effective population in the next 10 years (expected life span of the technology) and discounted by a rate of 4%. The Dutch effective population was calculated based on a yearly incidence of 1,743 HER2 positive early breast cancer patients eligible for chemotherapy (based on an annual incidence of 14,070 x 17.7% HER2+ x 70% eligible for chemotherapy). [22]

## RESULTS

In the real world scenario and the guideline scenario the trastuzumab treated patient group proved to be more expensive than the group of patients treated with chemotherapy alone. Costs were €243,216 and €239,657 for trastuzumab and no trastuzumab for the real world scenario, €224,443 and €218,948 for the guideline scenario (incremental cost of €3560 and €5495, respectively). However, in the trial scenario the no trastuzumab group was more expensive € 253,666 *versus* €265,116 (incremental cost of -€11,451). This was also the case for effectiveness in the three scenarios. The incremental QALYs of the real world and the guideline scenarios were 0.827 and 0.861 respectively, while the incremental QALY of the trial scenario was 0.993. Comparing these groups resulted in ICERs of €4304, €6382 and dominance, corresponding to the real world, guideline and trial scenarios. These base case results show that for the real world scenario and the guideline scenario trastuzumab is both more effective (LY and QALY gain) and more costly. In the trial scenario trastuzumab was less costly and more effective (Table 4).

**Table 4:** Base case results for all three scenarios

	Trastuzumab			No trastuzumab			
	Costs	LYs	QALYs	Costs	LYs		QALYs
	(95% CI)	(95% CI)	(95% CI)	(95% CI)	(95% CI)		(95% CI)
**Real world scenario**							
Trastuzumab treatment^a^	€ 31,061			€ 0			
	(€29,241 - €32,950)			(€0 - €0)			
Cardiac event / monitoring	€ 448		-0.016	€ 0			-0.003
	(€413 - €484)		(-0.024 - -0.009)	(€0 - €0)			(-0.005 - -0.001)
Disease free	€ 22,059	11.702	9.346	€ 20,629	10.173		8.115
	(€14,672 - €26,780)	(3.664 - 15.601)	(2.861 - 12.575)	(€13,818 - €25,232)	(2.978 - 14.200)		(2.301 - 11.416)
Local recurrence	€ 9,334	0.901	0.643	€ 7,532	0.720		0.514
	(€0 - €50,138)	(0.000 - 4.453)	(0.000 - 3.193)	(€0 - €40,739)	(0.000 - 3.627)		(0.000 - 2.624)
Distant metastases	€ 180,314	6.576	3.958	€ 211,496	7.438		4.476
	(€51,054 - €408,389)	(2.284 - 12.167)	(1.349 - 7.429)	(€67,569 - €455,225)	(2.849 - 12.974)		(1.705 - 7.954)
Total	€ 243,216	19.180	13.930	€ 239,657	18.331		13.103
	(€107,224 - €491,079)	(16.425 - 21.668)	(11.705 - 15.891)	(€91,498 - €497,968)	(15.264 - 21.112)		(10.769 - 15.239)
**Guideline scenario**							
Trastuzumab treatment^a^	€ 31,376			€ 0			
	(€29,565 - €33,271)			(€0 - €0)			
Cardiac event / monitoring	€ 449		-0.016	€ 0			-0.003
	(€415 - €485)		(-0.024 - -0.010)	(€0 - €0)			(-0.005 - -0.001)
Disease free	€ 22,398	12.045	9.618	€ 20,907	10.447		8.332
	(€20,052 - €25,169)	(10.611 - 13.492)	(8.387 - 10.878)	(€18,411 - €23,677)	(8.486 - 12.186)		(6.731 - 9.776)
Local recurrence	€ 3,236	0.334	0.238	€ 2,338	0.246		0.175
	(€78 - €12,343)	(0.006 - 1.226)	(0.005 - 0.874)	(€44 - €9,687)	(0.004 – 1.000)		(0.003 - 0.719)
Distant metastases	€ 166,984	6.132	3.688	€ 195,703	6.921		4.162
	(€66,592 - €318,118)	(2.967 - 9.186)	(1.757 - 5.631)	(€78,795 - €377,394)	(3.347 - 10.475)		(1.963 - 6.449)
Total	€ 224,443	18.511	13.527	€ 218,948	17.613		12.666
	(€124094 - €376,989)	(15.543 - 21.252)	(11.651 - 15.381)	(€102,933 - €400,182)	(14.357 - 20.644)		(10.593 - 14.715)

	Trastuzumab Costs (95% CI)			No Trastuzumab				
		Lys (95% CI)	QALYs (95% CI)	Costs (95%)		Lys (95% CI)	QALYs (95% CI)	
**Trial scenario**								
Trastuzumab treatment^a^	€ 30,564			€ 0				
	(€28,753 - €32,478)			(€0 - €0)				
Cardiac event / monitoring	€ 439		-0.004	€ 0				-0.001
	(€405 - €474)		(-0.006 - -0.003)	(€0 - €0)				(-0.001 - 0.000)
Disease free	€ 22,078	12.172	9.723	€ 19,776	9.996			7.974
	(€19,794 - €24,774)	(11.022 - 13.358)	(8.714 - 10.787)	(€17,319 - €22,551)	(8.199 - 11.718)			(6.507 - 9.405)
Local recurrence	€ 0	0.000	0.000	€ 0	0.000			0.000
	(€0 - €0)	(0.000 - 0.000)	(0.000 - 0.000)	(€0 - €0)	(0.000 - 0.000)			(0.000 - 0.000)
Distant metastases	€ 200,585	7.275	4.379	€ 245,341	8.523			5.131
	(€117,747 - €337,277)	(6.022 - 8.497)	(3.429 - 5.358)	(€140,871 - €416,862)	(6.778 - 10.307)			(3.883 - 6.485)
Total	€ 253,666	19.448	14.098	€ 265,116	18.519			13.104
	(€170,975 - €389,649)	(18.992 - 19.892)	(13.223 - 14.933)	(€160,895 - €437,653)	(17.515 - 19.308)			(12.009 - 14.145)

Incremental analyses							
	Trastuzumab		No trastuzumab		Increment		ICER
	Costs	QALYs	Costs	QALYs	Costs	QALYs	
**Real world scenario**	€ 243,216	13.930	€ 239,657	13.103	€ 3,560	0.827	€ 4,304
**Guideline scenario**	€ 224,443	13.527	€ 218,948	12.666	€ 5,495	0.861	€ 6,382
**Trial scenario**	€ 253,666	14.098	€ 265,116	13.104	-€11,451	0.993	Dominance

^0a^Trastuzumab treatment costs includes HER2 testing costs

In all three scenarios, the incremental costs could be credited to a high cost of treatment of distant metastases in the no trastuzumab group compared to the trastuzumab group. The incremental costs associated with the local recurrence health state were higher in the real world and guideline scenarios compared to the trial scenario. The trial scenario had no costs related to the local recurrence health state. This was caused by the fact that LR is calculated by 1-DFS and 1-DM which in this case is 0%. In our trial scenario 1-DFS < 1-DM (Table 2).

Similarly to the incremental cost, the incremental QALYs and LYs could be attributed to the higher number of QALYs and LYs generated in the distant metastases health state.

In the one-way sensitivity analyses the ICERs were sensitive to the duration of the treatment effect (Table 5). In addition, Table 6 indicates that assuming treatment dependent health state costs would result in dominance of trastuzumab in all three scenarios.

**Table 5:** Sensitivity analysis; assuming 4y treatment effect duration (instead of 8y) - probabilistic

	Trastuzumab		No trastuzumab		Increment		ICER
	Costs	QALYs	Costs	QALYs	Costs	QALYs	
Real world scenario	€ 242,295	13.938	€ 226,954	13.554	€ 15,341	0.384	€ 39,934
Guideline scenario	€ 226,014	13.545	€ 210,056	13.142	€ 15,957	0.402	€ 39,655
Trial scenario	€ 253,494	14.098	€ 249,955	13.547	€ 3,539	0.552	€ 6,412

**Table 6:** Sensitivity analysis; assuming treatment dependent DM health state costs - probabilistic

	Trastuzumab		No trastuzumab		Increment		ICER
	Costs	QALYs	Costs	QALYs	Costs	QALYs	
Real world scenario	€ 176,541	13.927	€ 315,715	13.097	-€ 139,175	0.830	Dominance
Guideline scenario	€ 165,256	13.551	€ 293,593	12.686	-€ 128,337	0.865	Dominance
Trial scenario	€ 181,133	14.095	€ 357,625	13.103	-€ 176,492	0.993	Dominance

**Figure 2:**
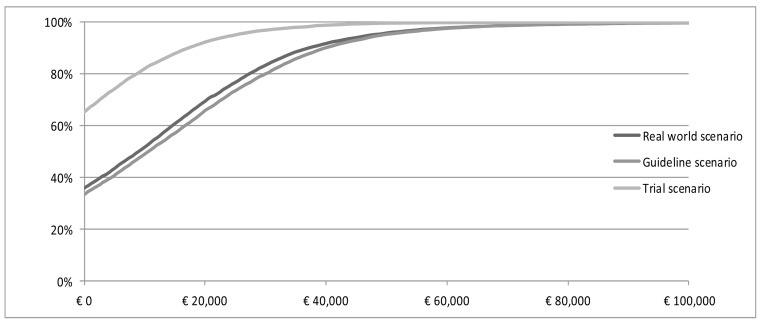
CEAC Cost-effectiveness acceptability curve of trastuzumab in real world scenario, guideline scenario and trial scenario.

At a willingness to pay (WTP) threshold of €80,000 per QALY gained, the probability that trastuzumab treatment is cost-effective is 99% or higher in all three scenarios (Figure 2). The expected value of perfect information (EVPI) analysis, at a threshold of €80,000 per QALY, amounts to €477,766, €486,041 and €1,346, respectively in the real world, guideline and trial scenario.

## DISCUSSION

In this study, we determined the costs and cost-effectiveness of one year of adjuvant trastuzumab treatment in early HER2-positive breast cancer *versus* chemotherapy alone. We assessed three scenarios. We found that the ICER in the real world scenario was €4,304 per QALY gained, in the guideline scenario the ICER was €6,382 per QALY gained and finally the trial scenario showed dominance for trastuzumab. Based on these results, we conclude that trastuzumab in early breast cancer is a cost-effective intervention in all three scenarios in the Netherlands. Very little to no uncertainty was seen.

Reports from several countries have shown that adjuvant trastuzumab in early breast cancer patients is cost-effective. Previously, two reviews showed modelled cost-effectiveness analyses based on data from clinical trials in patients with HER2-positive early breast cancer, treated with adjuvant trastuzumab. Our study is one of the few studies that included real world data in a cost-effectiveness analysis.

In our results, the trial scenario showed dominance, while even though the real world scenario showed trastuzumab to be cost effective, there was no dominance. This difference was due to higher cost in the distant metastases group in the trastuzumab group *versus* the no trastuzumab group. Before the introduction of trastuzumab, HER2 positive patients with metastatic disease had a very poor prognosis, the introduction of trastuzumab made the prognosis better, but was also more costly.

The systematic review from Chan et al. selected thirteen articles that included cost-effectiveness analyses of 10 countries. The cost-effectiveness ratios ranged from $5020/QALY to $134,610/QALY. Most studies reported a favorable cost-effectiveness. [23] Mc-Keage et al also performed a review, this study reported that trastuzumab was cost-effective from a healthcare payer or societal perspective in several countries. Incremental costs per QALY or life-year gained with trastuzumab administered subsequent to or concurrent with chemotherapy compared with chemotherapy alone were consistently within accepted local thresholds for cost effectiveness. [24]

Our trial scenario was based on the HERA trial. We based our DFS data on the 8-year FU and our DM data from the 2 year FU, since the 8year FU did not mention DM data. Reviews have shown that trastuzumab is considered cost-effective when using the HERA trial data, but also when using other trials including FinHER trial, NSABP B-31 and NCCTG N9831trials. For example Dedes et al., a model-based study from Swiss healthcare perspective, showed that adjuvant trastuzumab proved to be cost-effective based on the data of the HERA trial and based on the data of the FinHER study. [2] Garisson et al. concluded the same based on data of the NSABP B-31 and NCCTG N9831trials. [3] Likewise cost-effectiveness of trastuzumab was seen in different (developed) countries, next to Switzerland, also USA (ICER of 39,982 US dollars per QALY) [4], Italy (ICER of 14,861 US dollars) [25] and Norway (estimated ICER of €44,934 per QALY) [26]. In a Belgian study, a HERA trial scenario was compared to a FinHER scenario. One year trastuzumab was shown to be cost-effective although never cost saving due to the higher initial treatment costs, whereas the 9-week FinHER regimen was cost saving. [6] So, most studies suggest that trastuzumab may be cost-effective for treatment of early breast cancer in a 1-year treatment regimen, based on the frequently proposed threshold of 50.000 US dollar / QALY. [27, 28] This is comparable to the proposed Dutch threshold of €80,000.

To our knowledge, only one study discussed real world cost-effectiveness of trastuzumab. The study of Hedden et al. indicated that trastuzumab is cost-effective in a real world setting. In this Canadian context the cost per QALY gained was $13,095. [29]

Differences in ICER between the diverse studies could be explained by country-specific differences in health care systems, costs, modeling concepts and underlying assumptions. This underpins the necessity of country-specific cost-effectiveness analysis. Another argument for different ICERs between studies is the fact that the longer the follow up, the more cross over there is in patients that were still treated with trastuzumab, while originally at the start of the clinical trials these patient were included in the no trastuzumab group.

The 8-year follow up data from the HERA trial showed that the trastuzumab *versus* no trastuzumab groups were contaminated by a 52.1% crossover. [13] On the other hand, real world data is virtually always biased by confounding by indication, and hence any estimate of comparative effectiveness. In our study we used the relative effectiveness from the trials, therefore there could be no confounding by indication. If we had used relative effect of the real world data we would have had confounding by indication. As a result, the estimation of (longer-term) comparative effectiveness is challenged, whether trial data or real world data are used. Massive crossover in trials may lead to an underestimation of treatment effect, while confounding by indication is mostly thought to overestimate treatment effect, and methods are developed to correct for both types of biases. [30] As all three scenarios based comparative effectiveness on the HERA trial, the reported ICERs may be overestimations. In health policy decision making trial and real world evidence are complementary, and further research should focus on ways to combine and handle bias in both sources of evidence.

Our study was unique in comparing real world data to a trial data and a guideline scenario. Selecting patients according to the 2005 guideline (guideline scenario) leads to the highest ICER (€ 6,382), and both the real world and the guideline scenario result in higher ICERs than the trial scenario. These results confirm that analyses based on data from clinical trials contain the most beneficial scenario. In addition, a trend is seen that when a breakthrough product is released, there is also a fast implementation, causing the real world to be a step ahead of the guidelines. In this study the real world scenario is more cost-effective than the guideline scenario.

In conclusion, adjuvant trastuzumab in the real world can be considered cost-effective in all three scenarios, but most cost-effective when all input is based on trial data.
